# Supplementary material for: Dose-Intense Cisplatin-Based Neoadjuvant Chemotherapy Increases Survival in Advanced Cervical Cancer: An Up-to-Date Meta-Analysis
Source: Cancers (Basel). 2022 Feb 8;14(3):842. doi: 10.3390/cancers14030842 (PMC8834199; doi:10.3390/cancers14030842)
Supplement: Supplementary file 1 [file cancers-14-00842-s001.zip › cancers-1512614-Supplementary Figures.pdf]

Supplementary Figure legends

Supplementary Figure S1: Funnel plot of risk of publication bias in randomized clinical trials published between 1991 and 2019 assessing PFS in locally-advanced cervical cancers. The distribution of plots shows no publication bias (*P* = 0.79).

Supplementary Figure S2: Forest plot for OS. The analysis of all pooled studies shows no significant benefit of using neoadjuvant chemotherapy, RR = 0.97 (95% CI, 0.9 to 1.05). There is a significant heterogeneity between studies, $I^2 = 0.45$, $P = 0.01$.

Supplementary Figure S3: Forest plot for OS as function of cisplatin ≥ 72.5 mg/m^2/3-weeks. Neoadjuvant chemotherapy using dose-dense cisplatin ≥ 72.5 mg/m^2/3-weeks improves significantly overall survival: RR for fixed effect = 0.87 (95% CI, 0.76 to 0.98). There is no heterogeneity between studies in each sub-group, $I^2 = 36\%$, $P = 0.11$.

Supplementary Figure S4: Forest plot for OS as function of cisplatin ≥ 75 mg/m^2/3-weeks with local treatment subgroup as surgery. A dose-dense ≥ 75 mg/m^2/3-weeks was significantly associated with a better survival and a RR of 0.78 [0.63-0.97, with no heterogeneity between studies in each sub-group, $I^2 = 19\%$, $P = 0.28$

Supplementary Figure S5: Forest plot for OS as function of cisplatin ≥ 105 mg/m^2/3-weeks with local treatment subgroup as radiotherapy. A dose-dense ≥ 105 mg/m^2/3-weeks was significantly associated with a better survival and a RR of 0.75 [0.59-0.96], with no heterogeneity between studies in each sub-group, $P > 0.05$

Supplementary Figure S6: Forest plot for OS as function of triplet chemotherapy. The sub-group analysis does not evidence any significant benefit of using a triplet regimen during neoadjuvant chemotherapy for the treatment of locally advanced cervical cancer, RR = 0.97 (95% CI, 0.83 to 1.13), with a significant heterogeneity between studies in each sub-group, $I^2 = 57\%$, $P < 0.01$.

Supplementary Figure S7: Forest plot for OS as function of chemotherapy duration. The sub-group analysis shows an unsignificant trend for a benefit of neoadjuvant chemotherapy duration less than

6 weeks, RR = 0.91 (95% CI, 0.80 to 1.04), but with a significant heterogeneity between studies in this sub-group, $I^2 = 49\%$, $P < 0.05$.

Supplementary Figure S8: Forest plot for PFS as function of cisplatin ≥ 72.5 mg/m^2/3-weeks. Neoadjuvant chemotherapy using dose-dense cisplatin ≥ 72.5 mg/m^2/3-weeks improves significantly progression-free survival: RR for fixed effect = 0.86 (95% CI, 0.78 to 0.95). There is no heterogeneity between studies in each sub-group, $P = 0.43$.

Supplementary Figure S9: Forest plot for PFS as function of surgery as local treatment. The sub-group analysis shows an unsignificant trend for a PFS benefit of neoadjuvant chemotherapy followed by surgery: RR for fixed effect = 0.86 (95% CI, 0.78 to 0.95). There is no heterogeneity between studies in each sub-group, $P = 0.43$.

Supplementary Figure S10: Funnel plot of risk of publication bias in randomized clinical trials published between 1991 and 2019 assessing OS in locally advanced cervical cancer. The distribution of plots shows no publication bias ($P = 0.73$).

Supplementary Figure S11: Risk of bias assessments for the 22 studies included in a Cochrane review.

Supplementary Figure S12: Overall assessment of risk of bias : graph for the 22 studies included in this meta-analysis.

Supplementary Figure S13: Forest plot for OS as function of cisplatin ≥ 105 mg/m^2/3-weeks after exclusion two studies (Benedetti-Panici P 2002 and Chang TC 2000). Neoadjuvant chemotherapy using dose-dense cisplatin ≥ 105 mg/m2/3-weeks improves significantly overall survival: RR for fixed effect = 0.76 (95% CI, 0.60 to 0.97). There is no heterogeneity between studies in each sub-group, $I^2 = 50\%$, $P = 0.09$.

Supplementary Figure

Supplementary Figure S1

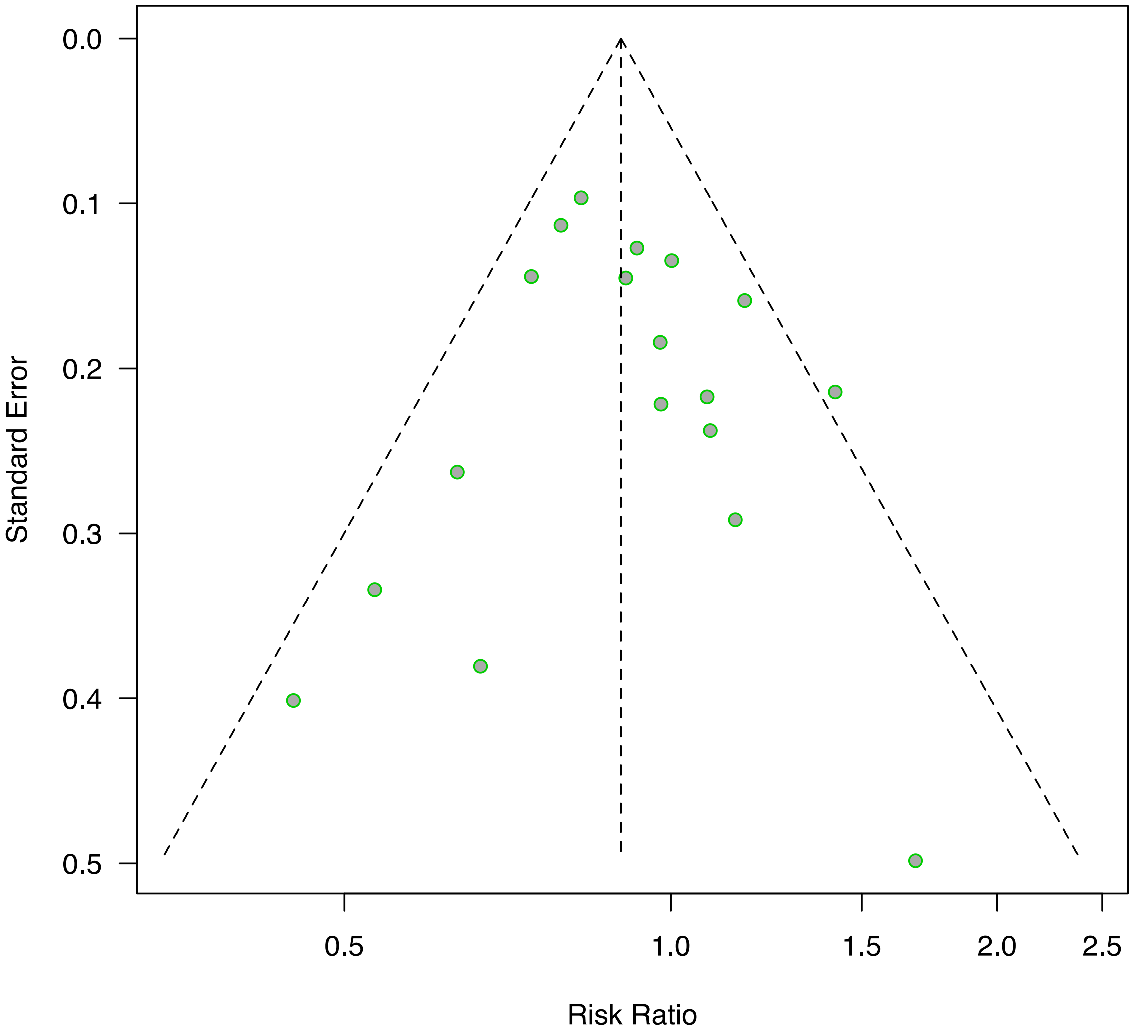

Supplementary Figure S2

Study	Experimental Events	Experimental Total	Control Events	Control Total	RR	95%-CI	Weight (fixed)	Weight (random)
da Costa SCS. et al 2019	20	55	13	52	1.45	[0.81; 2.61]	1.8%	2.5%
Yang Z et al. 2016	8	109	5	110	1.61	[0.55; 4.78]	0.7%	0.9%
Katsumata N et al. 2013	17	67	16	67	1.06	[0.59; 1.92]	2.2%	2.5%
Mossa B et al. 2010	47	159	44	129	0.87	[0.62; 1.22]	6.6%	5.2%
Chen H et al. 2008	20	72	31	70	0.63	[0.40; 0.99]	4.3%	3.6%
Eddy GL et al. 2007	53	145	51	143	1.02	[0.75; 1.39]	7.0%	5.8%
Cai HB et al. 2006	8	52	13	54	0.64	[0.29; 1.41]	1.7%	1.5%
Tabata T et al. 2003	18	32	14	29	1.17	[0.72; 1.89]	2.0%	3.3%
Benedetti-Panici P et al. 2002	88	227	101	214	0.82	[0.66; 1.02]	14.1%	7.8%
Chang TC et al. 2000	15	68	12	52	0.96	[0.49; 1.86]	1.8%	2.0%
Herod J et al. 2000	65	86	60	86	1.08	[0.90; 1.30]	8.1%	8.6%
Symonds RP et al. 2000	66	100	68	104	1.01	[0.83; 1.23]	9.0%	8.2%
Kumar L et al. 1998	49	88	34	85	1.39	[1.01; 1.92]	4.7%	5.6%
Sardi JE et al. 1998	30	73	33	74	0.92	[0.63; 1.34]	4.4%	4.7%
Sardi JE et al. 1997	19	102	32	103	0.60	[0.36; 0.99]	4.3%	3.2%
Sardi JE et al. 1996	25	54	41	54	0.61	[0.44; 0.84]	5.6%	5.5%
Sundfor K et al. 1996	29	47	28	47	1.04	[0.75; 1.43]	3.8%	5.5%
Kigawa J et al. 1996	10	25	15	25	0.67	[0.37; 1.19]	2.0%	2.6%
Tattersall MH et al. 1995	38	129	28	131	1.38	[0.90; 2.10]	3.8%	4.0%
Chauvergne J et al. 1993	45	75	45	76	1.01	[0.78; 1.32]	6.1%	6.7%
Tattersall MH et al. 1992	20	34	18	37	1.21	[0.78; 1.87]	2.3%	3.9%
Souhami L et al. 1991	30	39	31	52	1.29	[0.97; 1.71]	3.6%	6.3%
**Fixed effect model**		**1838**		**1794**	**0.97**	**[0.90; 1.05]**	**100.0%**	**--**
**Random effects model**					**0.98**	**[0.88; 1.09]**	**--**	**100.0%**

Heterogeneity: $I^2 = 45\%$, $\tau^2 = 0.0247$, $p = 0.01$

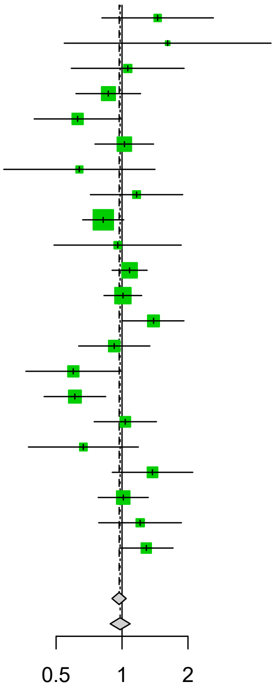

Supplementary Figure S3

Study	Experimental Events	Experimental Total	Control Events	Control Total	RR	95%-CI	Weight
**Platinum dose intensity ≥ 72.5 mg/m2/3-week = No**							
da Costa SCS. et al 2019	20	55	13	52	1.45	[0.81; 2.61]	2.5%
Yang Z et al. 2016	8	109	5	110	1.61	[0.55; 4.78]	0.9%
Katsumata N et al. 2013	17	67	16	67	1.06	[0.59; 1.92]	2.5%
Mossa B et al. 2010	47	159	44	129	0.87	[0.62; 1.22]	5.2%
Tabata T et al. 2003	18	32	14	29	1.17	[0.72; 1.89]	3.3%
Herod J et al. 2000	65	86	60	86	1.08	[0.90; 1.30]	8.6%
Kumar L et al. 1998	49	88	34	85	1.39	[1.01; 1.92]	5.6%
Kigawa J et al. 1996	10	25	15	25	0.67	[0.37; 1.19]	2.6%
Tattersall MH et al. 1995	38	129	28	131	1.38	[0.90; 2.10]	4.0%
Tattersall MH et al. 1992	20	34	18	37	1.21	[0.78; 1.87]	3.9%
Souhami L et al. 1991	30	39	31	52	1.29	[0.97; 1.71]	6.3%
**Random effects model**		**823**		**803**	**1.15**	**[1.02; 1.28]**	**45.4%**
Heterogeneity: $I^2 = 3\%$, $\tau^2 = 0.0012$, $p = 0.41$							
**Platinum dose intensity ≥ 72.5 mg/m2/3-week = Yes**							
Chen H et al. 2008	20	72	31	70	0.63	[0.40; 0.99]	3.6%
Eddy GL et al. 2007	53	145	51	143	1.02	[0.75; 1.39]	5.8%
Cai HB et al. 2006	8	52	13	54	0.64	[0.29; 1.41]	1.5%
Benedetti-Panici P et al. 2002	88	227	101	214	0.82	[0.66; 1.02]	7.8%
Chang TC et al. 2000	15	68	12	52	0.96	[0.49; 1.86]	2.0%
Symonds RP et al. 2000	66	100	68	104	1.01	[0.83; 1.23]	8.2%
Sardi JE et al. 1998	30	73	33	74	0.92	[0.63; 1.34]	4.7%
Sardi JE et al. 1997	19	102	32	103	0.60	[0.36; 0.99]	3.2%
Sardi JE et al. 1996	25	54	41	54	0.61	[0.44; 0.84]	5.5%
Sundfor K et al. 1996	29	47	28	47	1.04	[0.75; 1.43]	5.5%
Chauvergne J et al. 1993	45	75	45	76	1.01	[0.78; 1.32]	6.7%
**Random effects model**		**1015**		**991**	**0.87**	**[0.76; 0.98]**	**54.6%**
Heterogeneity: $I^2 = 36\%$, $\tau^2 = 0.0153$, $p = 0.11$							
**Random effects model**		**1838**		**1794**	**0.98**	**[0.88; 1.09]**	**100.0%**
Heterogeneity: $I^2 = 45\%$, $\tau^2 = 0.0247$, $p = 0.01$							
Residual heterogeneity: $I^2 = 23\%$, $p = 0.17$							

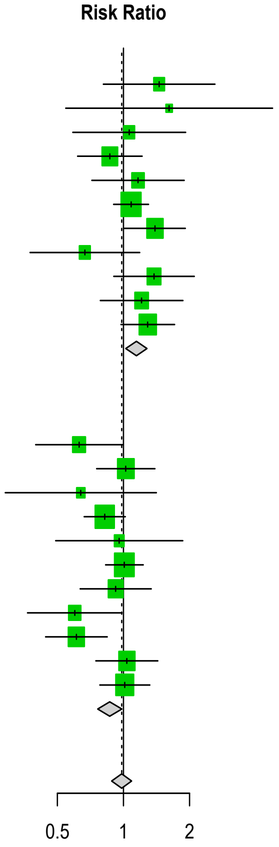

Supplementary Figure S4

Study	Experimental Events	Experimental Total	Control Events	Control Total	RR	95%-CI	Weight
**Platinum dose intensity ≥ 75 mg/m2/3-week = No**							
Yang Z et al. 2016	8	109	5	110	1.61	[0.55; 4.78]	2.5%
Katsumata N et al. 2013	17	67	16	67	1.06	[0.59; 1.92]	8.1%
Mossa B et al. 2010	47	159	44	129	0.87	[0.62; 1.22]	24.7%
**Fixed effect model**		335		306	**0.97**	**[0.73; 1.28]**	**35.3%**
Heterogeneity: $I^2 = 0\%$, $\tau^2 = 0$, $p = 0.51$							
**Platinum dose intensity ≥ 75 mg/m2/3-week = Yes**							
Chen H et al. 2008	20	72	31	70	0.63	[0.40; 0.99]	16.0%
Eddy GL et al. 2007	53	145	51	143	1.02	[0.75; 1.39]	26.1%
Cai HB et al. 2006	8	52	13	54	0.64	[0.29; 1.41]	6.5%
Sardi JE et al. 1997	19	102	32	103	0.60	[0.36; 0.99]	16.2%
**Fixed effect model**		371		370	**0.78**	**[0.63; 0.97]**	**64.7%**
Heterogeneity: $I^2 = 43\%$, $\tau^2 = 0.0422$, $p = 0.16$							
**Fixed effect model**		**706**		**676**	**0.85**	**[0.71; 1.01]**	**100.0%**

Heterogeneity: $I^2 = 19\%$, $\tau^2 = 0.0141$, $p = 0.28$

Test for subgroup differences: $\chi^2_1 = 1.33$, df = 1 ($p = 0.25$)

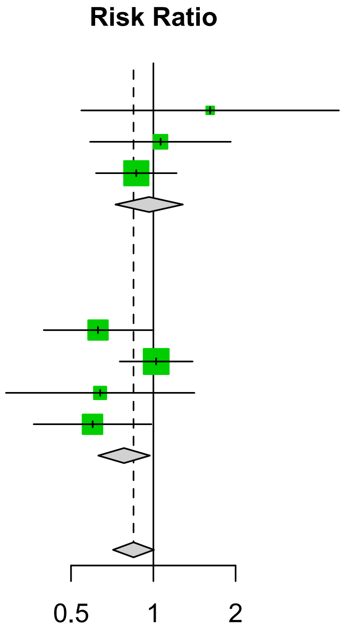

Supplementary Figure S5

Study	Experimental Events	Experimental Total	Control Events	Control Total	RR	95%-CI	Weight
**Platinum dose intensity ≥ 105 mg/m2/3-week = No**							
Tabata T et al. 2003	18	32	14	29	1.17	[0.72; 1.89]	3.7%
Herod J et al. 2000	65	86	60	86	1.08	[0.90; 1.30]	15.2%
Symonds RP et al. 2000	66	100	68	104	1.01	[0.83; 1.23]	16.9%
Kumar L et al. 1998	49	88	34	85	1.39	[1.01; 1.92]	8.8%
Sundfor K et al. 1996	29	47	28	47	1.04	[0.75; 1.43]	7.1%
Tattersall MH et al. 1995	38	129	28	131	1.38	[0.90; 2.10]	7.1%
Chauvergne J et al. 1993	45	75	45	76	1.01	[0.78; 1.32]	11.3%
Tattersall MH et al. 1992	20	34	18	37	1.21	[0.78; 1.87]	4.4%
Souhami L et al. 1991	30	39	31	52	1.29	[0.97; 1.71]	6.7%
**Fixed effect model**		**630**		**647**	**1.14**	**[1.04; 1.26]**	**81.3%**
Heterogeneity: $I^2 = 0\%$, $\tau^2 = 0$, $p = 0.65$							
**Platinum dose intensity ≥ 105 mg/m2/3-week = Yes**							
Sardi JE et al. 1998	30	73	33	74	0.92	[0.63; 1.34]	8.3%
Sardi JE et al. 1996	25	54	41	54	0.61	[0.44; 0.84]	10.4%
**Fixed effect model**		**127**		**128**	**0.75**	**[0.59; 0.96]**	**18.7%**
Heterogeneity: $I^2 = 63\%$, $\tau^2 = 0.0549$, $p = 0.10$							
**Fixed effect model**		**757**		**775**	**1.07**	**[0.98; 1.17]**	**100.0%**
Heterogeneity: $I^2 = 47\%$, $\tau^2 = 0.0197$, $p = 0.04$							
Test for subgroup differences: $\chi^2_1 = 9.78$, df = 1 ($p < 0.01$)							

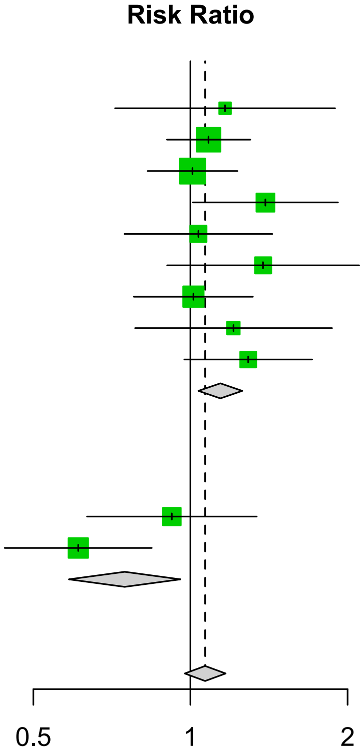

Supplementary Figure S6

Study	Experimental Events	Experimental Total	Control Events	Control Total	RR	95%-CI	Weight
**Triplet chemotherapy = No**							
da Costa SCS. et al 2019	20	55	13	52	1.45	[0.81; 2.61]	2.5%
Yang Z et al. 2016	8	109	5	110	1.61	[0.55; 4.78]	0.9%
Eddy GL et al. 2007	53	145	51	143	1.02	[0.75; 1.39]	5.8%
Cai HB et al. 2006	8	52	13	54	0.64	[0.29; 1.41]	1.5%
Benedetti-Panici P et al. 2002	88	227	101	214	0.82	[0.66; 1.02]	7.8%
Symonds RP et al. 2000	66	100	68	104	1.01	[0.83; 1.23]	8.2%
Sundfor K et al. 1996	29	47	28	47	1.04	[0.75; 1.43]	5.5%
Kigawa J et al. 1996	10	25	15	25	0.67	[0.37; 1.19]	2.6%
Tattersall MH et al. 1995	38	129	28	131	1.38	[0.90; 2.10]	4.0%
Random effects model		889		880	0.99	[0.86; 1.14]	38.8%
Heterogeneity: $I^2 = 25\%$, $\tau^2 = 0.0111$, $p = 0.22$							
**Triplet chemotherapy = Yes**							
Katsumata N et al. 2013	17	67	16	67	1.06	[0.59; 1.92]	2.5%
Mossa B et al. 2010	47	159	44	129	0.87	[0.62; 1.22]	5.2%
Chen H et al. 2008	20	72	31	70	0.63	[0.40; 0.99]	3.6%
Tabata T et al. 2003	18	32	14	29	1.17	[0.72; 1.89]	3.3%
Chang TC et al. 2000	15	68	12	52	0.96	[0.49; 1.86]	2.0%
Herod J et al. 2000	65	86	60	86	1.08	[0.90; 1.30]	8.6%
Kumar L et al. 1998	49	88	34	85	1.39	[1.01; 1.92]	5.6%
Sardi JE et al. 1998	30	73	33	74	0.92	[0.63; 1.34]	4.7%
Sardi JE et al. 1997	19	102	32	103	0.60	[0.36; 0.99]	3.2%
Sardi JE et al. 1996	25	54	41	54	0.61	[0.44; 0.84]	5.5%
Chauvergne J et al. 1993	45	75	45	76	1.01	[0.78; 1.32]	6.7%
Tattersall MH et al. 1992	20	34	18	37	1.21	[0.78; 1.87]	3.9%
Souhami L et al. 1991	30	39	31	52	1.29	[0.97; 1.71]	6.3%
Random effects model		949		914	0.97	[0.83; 1.13]	61.2%
Heterogeneity: $I^2 = 57\%$, $\tau^2 = 0.0406$, $p < 0.01$							
**Random effects model**		**1838**		**1794**	**0.98**	**[0.88; 1.09]**	**100.0%**
Heterogeneity: $I^2 = 45\%$, $\tau^2 = 0.0247$, $p = 0.01$							
Residual heterogeneity: $I^2 = 48\%$, $p < 0.01$							

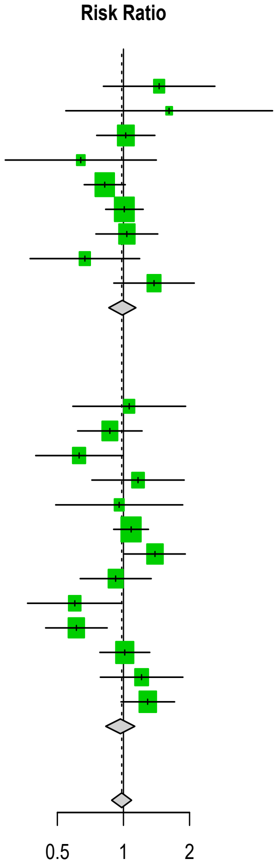

Supplementary Figure S7

Study	Experimental Events	Experimental Total	Control Events	Control Total	RR	95%-CI	Weight
**Chemotherapy ≤ 6 weeks = No**							
da Costa SCS. et al 2019	20	55	13	52	1.45	[0.81; 2.61]	2.5%
Mossa B et al. 2010	47	159	44	129	0.87	[0.62; 1.22]	5.2%
Tabata T et al. 2003	18	32	14	29	1.17	[0.72; 1.89]	3.3%
Sundfor K et al. 1996	29	47	28	47	1.04	[0.75; 1.43]	5.5%
Tattersall MH et al. 1995	38	129	28	131	1.38	[0.90; 2.10]	4.0%
Tattersall MH et al. 1992	20	34	18	37	1.21	[0.78; 1.87]	3.9%
Souhami L et al. 1991	30	39	31	52	1.29	[0.97; 1.71]	6.3%
**Random effects model**		**495**		**477**	**1.15**	**[1.00; 1.33]**	**30.8%**
Heterogeneity: $I^2 = 0\%$, $\tau^2 = 0$, $p = 0.53$							
**Chemotherapy ≤ 6 weeks = Yes**							
Yang Z et al. 2016	8	109	5	110	1.61	[0.55; 4.78]	0.9%
Katsumata N et al. 2013	17	67	16	67	1.06	[0.59; 1.92]	2.5%
Chen H et al. 2008	20	72	31	70	0.63	[0.40; 0.99]	3.6%
Eddy GL et al. 2007	53	145	51	143	1.02	[0.75; 1.39]	5.8%
Cai HB et al. 2006	8	52	13	54	0.64	[0.29; 1.41]	1.5%
Benedetti-Panici P et al. 2002	88	227	101	214	0.82	[0.66; 1.02]	7.8%
Chang TC et al. 2000	15	68	12	52	0.96	[0.49; 1.86]	2.0%
Herod J et al. 2000	65	86	60	86	1.08	[0.90; 1.30]	8.6%
Symonds RP et al. 2000	66	100	68	104	1.01	[0.83; 1.23]	8.2%
Kumar L et al. 1998	49	88	34	85	1.39	[1.01; 1.92]	5.6%
Sardi JE et al. 1998	30	73	33	74	0.92	[0.63; 1.34]	4.7%
Sardi JE et al. 1997	19	102	32	103	0.60	[0.36; 0.99]	3.2%
Sardi JE et al. 1996	25	54	41	54	0.61	[0.44; 0.84]	5.5%
Kigawa J et al. 1996	10	25	15	25	0.67	[0.37; 1.19]	2.6%
Chauvergne J et al. 1993	45	75	45	76	1.01	[0.78; 1.32]	6.7%
**Random effects model**		**1343**		**1317**	**0.91**	**[0.80; 1.04]**	**69.2%**
Heterogeneity: $I^2 = 49\%$, $\tau^2 = 0.0271$, $p = 0.02$							
**Random effects model**		**1838**		**1794**	**0.98**	**[0.88; 1.09]**	**100.0%**
Heterogeneity: $I^2 = 45\%$, $\tau^2 = 0.0247$, $p = 0.01$							
Residual heterogeneity: $I^2 = 39\%$, $p = 0.04$							

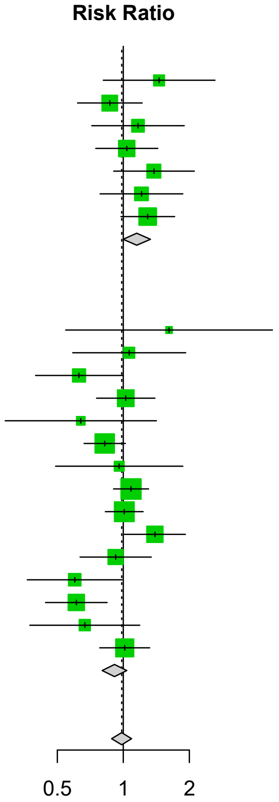

Supplementary Figure S8

Study	Experimental Events	Experimental Total	Control Events	Control Total	RR	95%-CI	Weight
**Platinum dose intensity ≥ 72.5 mg/m2/3-week = No**							
Costa SCS et al. 2019	30	55	20	52	1.42	[0.93; 2.16]	4.6%
Yang Z et al. 2016	10	109	6	110	1.68	[0.63; 4.47]	1.0%
Katsumata N et al. 2013	27	67	25	67	1.08	[0.71; 1.65]	4.5%
Mossa B et al. 2010	55	159	60	129	0.74	[0.56; 0.99]	8.1%
Tabata T et al. 2003	18	32	15	29	1.09	[0.68; 1.73]	3.9%
Kigawa J et al. 1996	8	25	15	25	0.53	[0.28; 1.03]	2.1%
Tattersall MH et al. 1995	53	129	46	131	1.17	[0.86; 1.60]	7.1%
Tattersall MH et al. 1992	18	34	20	37	0.98	[0.63; 1.51]	4.3%
Souhami L et al. 1991	22	39	30	52	0.98	[0.68; 1.40]	5.8%
**Random effects model**		**649**		**632**	**1.00**	**[0.84; 1.20]**	**41.4%**
Heterogeneity: I^2 = 37%, τ^2 = 0.0263, p = 0.12							
**Platinum dose intensity ≥ 72.5 mg/m2/3-week = Yes**							
Chen H et al. 2008	17	72	26	70	0.64	[0.38; 1.06]	3.3%
Eddy GL et al. 2007	63	145	62	143	1.00	[0.77; 1.30]	8.8%
Cai HB et al. 2006	9	52	14	54	0.67	[0.32; 1.41]	1.7%
Benedetti-Panici P et al. 2002	84	227	100	214	0.79	[0.63; 0.99]	10.7%
Chang TC et al. 2000	21	68	14	52	1.15	[0.65; 2.03]	2.7%
Symonds RP et al. 2000	62	100	78	104	0.83	[0.68; 1.00]	12.4%
Sardi JE et al. 1997	8	102	18	103	0.45	[0.20; 0.99]	1.5%
Sundfor K et al. 1996	30	47	33	47	0.91	[0.68; 1.21]	8.0%
Chauvergne J et al. 1993	45	75	49	76	0.93	[0.73; 1.19]	9.4%
**Random effects model**		**888**		**863**	**0.86**	**[0.78; 0.95]**	**58.6%**
Heterogeneity: I^2 = 0%, τ^2 = 0, p = 0.43							
**Random effects model**		**1537**		**1495**	**0.91**	**[0.82; 1.01]**	**100.0%**
Heterogeneity: I^2 = 28%, τ^2 = 0.0120, p = 0.13							
Test for subgroup differences: χ^2_1 = 2.29, df = 1 (p = 0.13)							

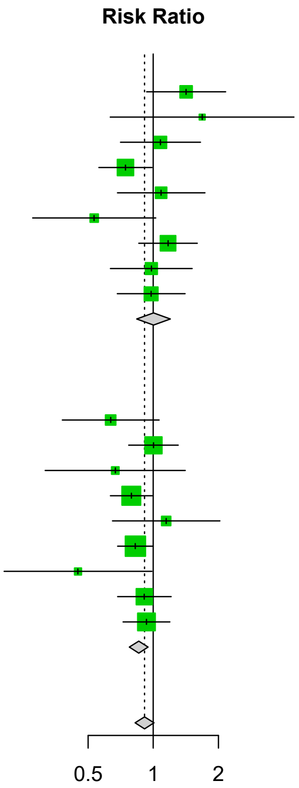

Supplementary Figure S9

Study	Experimental Events	Experimental Total	Control Events	Control Total	RR	95%-CI	Weight
**Protocol = NAC-RT vs RT**							
Tabata T et al. 2003	18	32	15	29	1.09	[0.68; 1.73]	4.1%
Symonds RP et al. 2000	62	100	78	104	0.83	[0.68; 1.00]	19.1%
Sundfor K et al. 1996	30	47	33	47	0.91	[0.68; 1.21]	10.1%
Tattersall MH et al. 1995	53	129	46	131	1.17	[0.86; 1.60]	8.6%
Chauvergne J et al. 1993	45	75	49	76	0.93	[0.73; 1.19]	12.6%
Tattersall MH et al. 1992	18	34	20	37	0.98	[0.63; 1.51]	4.7%
Souhami L et al. 1991	22	39	30	52	0.98	[0.68; 1.40]	6.6%
**Random effects model**		**456**		**476**	**0.93**	**[0.84; 1.04]**	**65.9%**
Heterogeneity: $I^2 = 0\%$, $\tau^2 = 0$, $p = 0.63$							
**Protocol = NAC-surgery vs surgery**							
Yang Z et al. 2016	10	109	6	110	1.68	[0.63; 4.47]	1.0%
Katsumata N et al. 2013	27	67	25	67	1.08	[0.71; 1.65]	4.9%
Mossa B et al. 2010	55	159	60	129	0.74	[0.56; 0.99]	10.2%
Chen H et al. 2008	17	72	26	70	0.64	[0.38; 1.06]	3.4%
Eddy GL et al. 2007	63	145	62	143	1.00	[0.77; 1.30]	11.4%
Cai HB et al. 2006	9	52	14	54	0.67	[0.32; 1.41]	1.7%
Sardi JE et al. 1997	8	102	18	103	0.45	[0.20; 0.99]	1.5%
**Random effects model**		**706**		**676**	**0.84**	**[0.67; 1.04]**	**34.1%**
Heterogeneity: $I^2 = 38\%$, $\tau^2 = 0.0311$, $p = 0.14$							
**Random effects model**		**1162**		**1152**	**0.91**	**[0.82; 1.00]**	**100.0%**
Heterogeneity: $I^2 = 11\%$, $\tau^2 = 0.0037$, $p = 0.33$							
Test for subgroup differences: $\chi^2_1 = 0.74$, df = 1 ($p = 0.39$)							

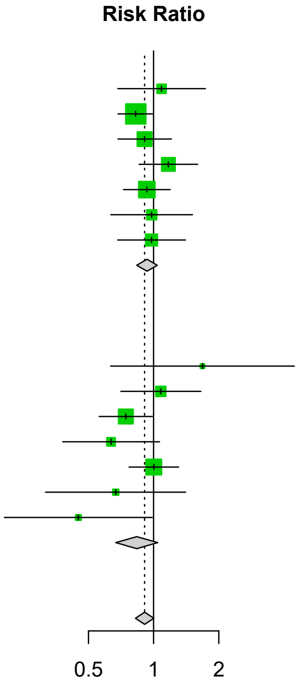

Supplementary Figure S10

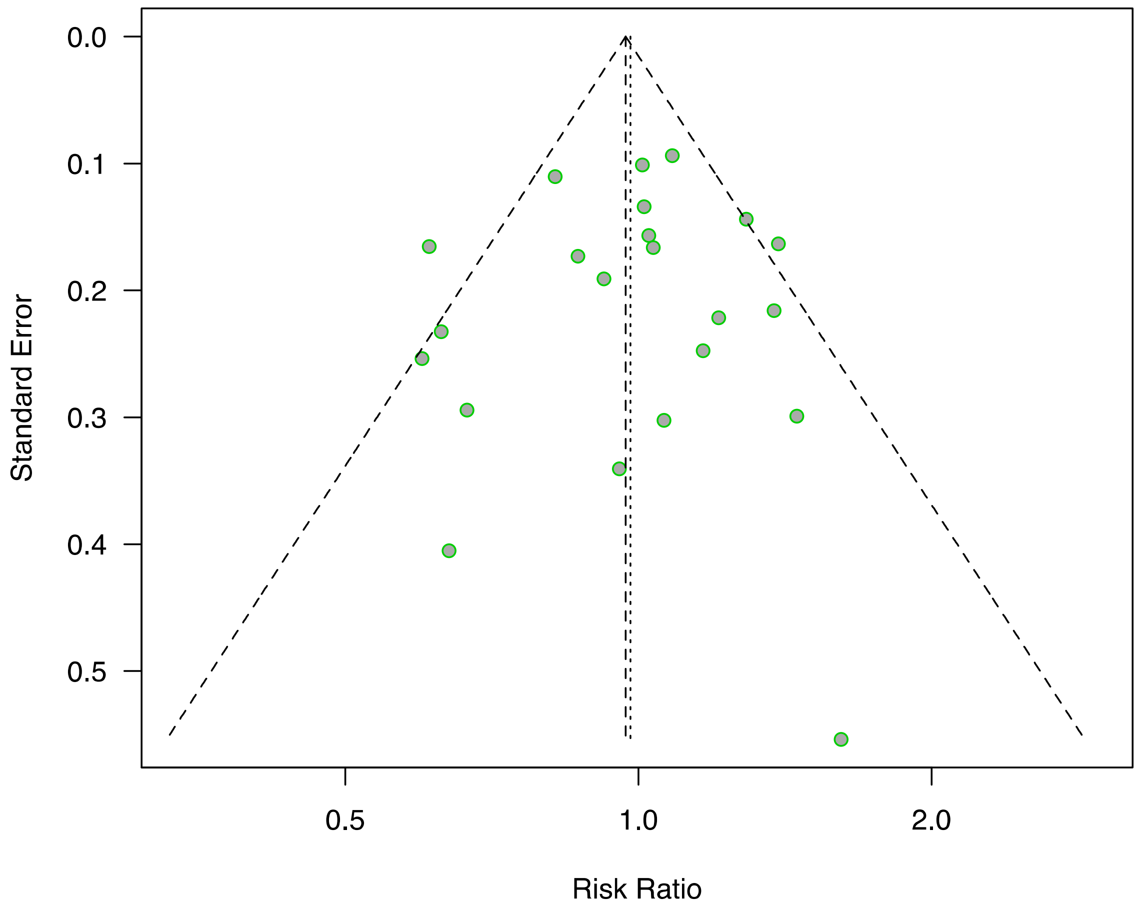

Supplementary Figure S11

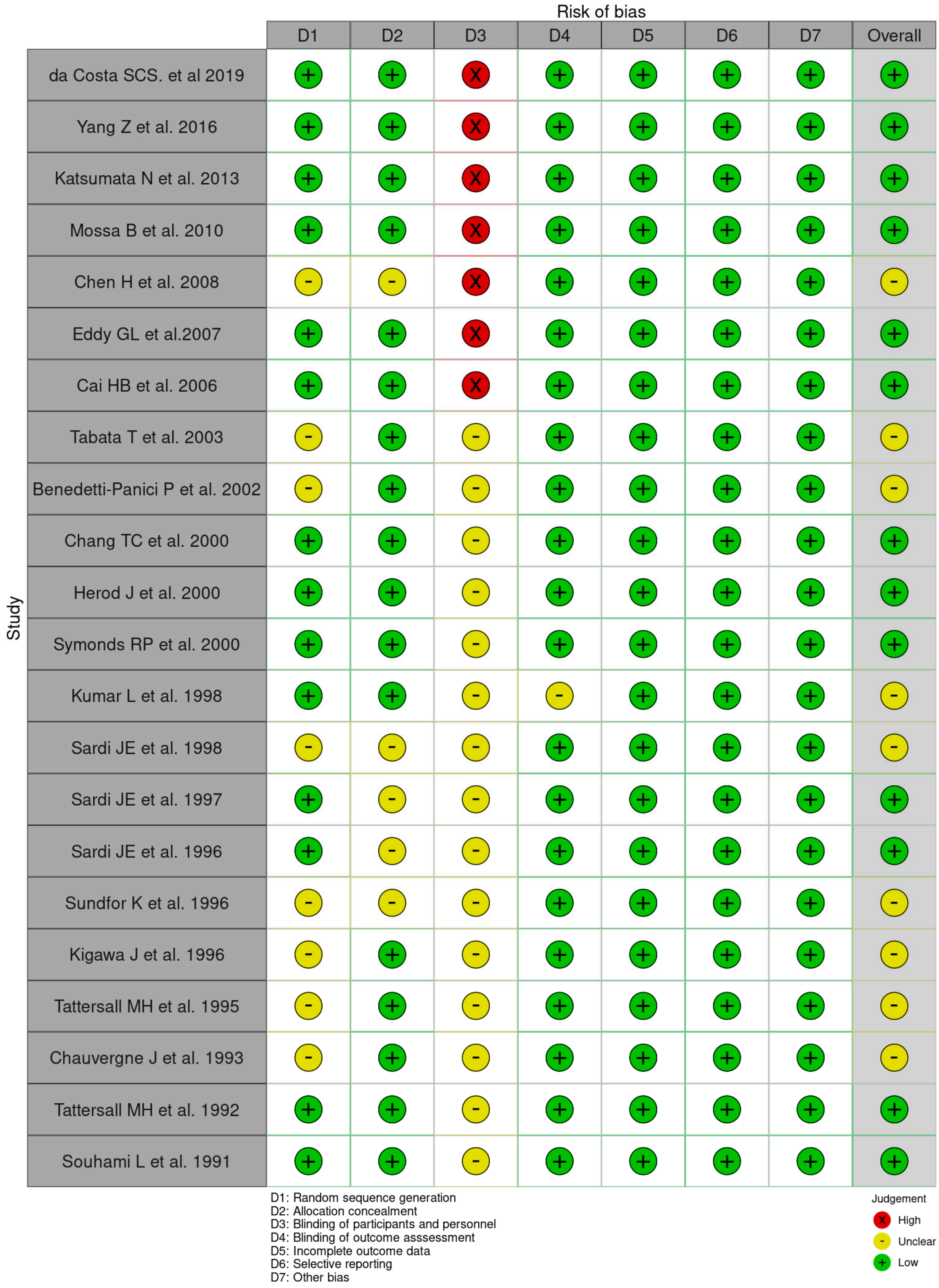

Supplementary Figure S12

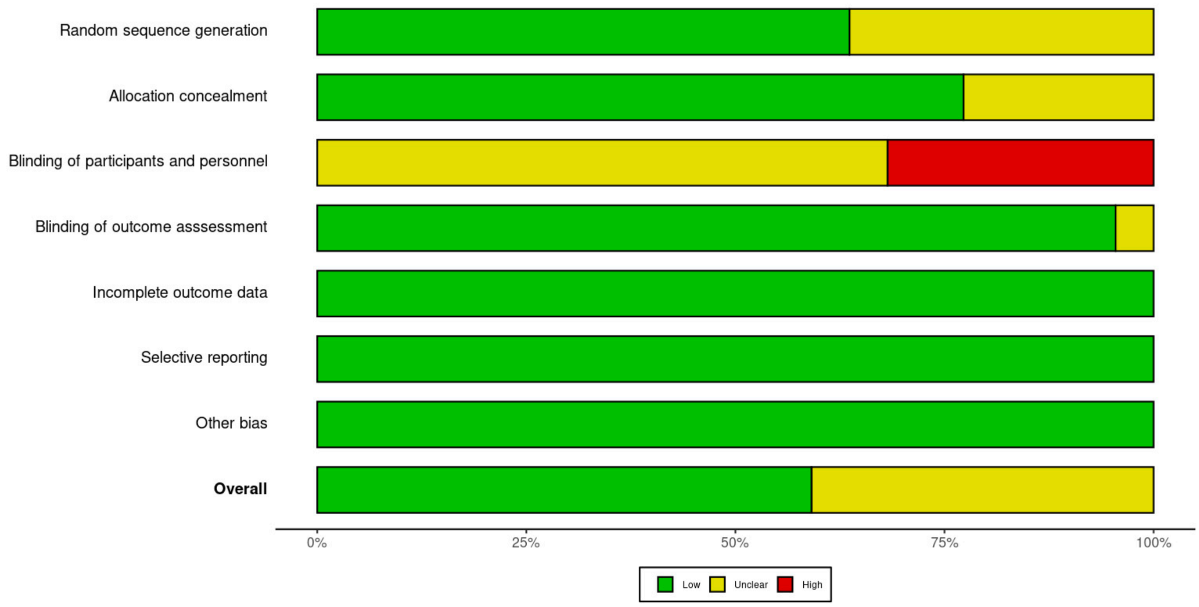

Supplementary Figure S13

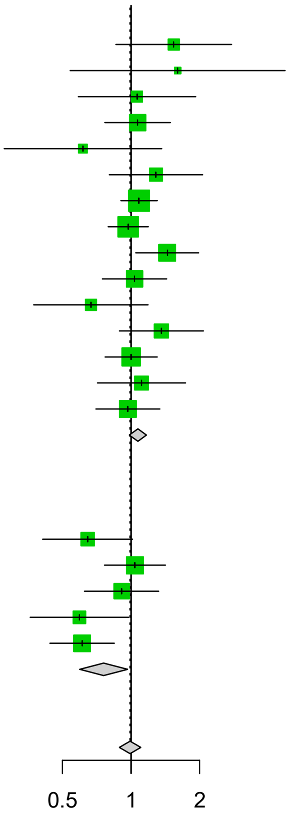

Study	Experimental Events	Experimental Total	Control Events	Control Total	RR	95%-CI	Weight
**Platinum dose intensity ≥ 105 mg/m2/3-week = No**							
da Costa SCS. et al 2019	20	52	13	52	1.54	[0.86; 2.76]	2.8%
Yang Z et al. 2016	8	110	5	110	1.60	[0.54; 4.74]	0.9%
Katsumata N et al. 2013	17	67	16	67	1.06	[0.59; 1.92]	2.7%
Mossa B et al. 2010	47	129	44	129	1.07	[0.77; 1.49]	6.0%
Cai HB et al. 2006	8	54	13	54	0.62	[0.28; 1.36]	1.7%
Tabata T et al. 2003	18	29	14	29	1.29	[0.80; 2.06]	3.8%
Herod J et al. 2000	65	86	60	86	1.08	[0.90; 1.30]	9.8%
Symonds RP et al. 2000	66	104	68	104	0.97	[0.79; 1.19]	9.2%
Kumar L et al. 1998	49	85	34	85	1.44	[1.05; 1.98]	6.3%
Sundfor K et al. 1996	29	47	28	47	1.04	[0.75; 1.43]	6.1%
Kigawa J et al. 1996	10	25	15	25	0.67	[0.37; 1.19]	2.8%
Tattersall MH et al. 1995	38	131	28	131	1.36	[0.89; 2.07]	4.4%
Chauvergne J et al. 1993	45	76	45	76	1.00	[0.77; 1.30]	7.5%
Tattersall MH et al. 1992	20	37	18	37	1.11	[0.71; 1.73]	4.2%
Souhami L et al. 1991	30	52	31	52	0.97	[0.70; 1.34]	6.2%
**Random effects model**		**1084**		**1084**	**1.07**	**[0.98; 1.17]**	**74.5%**
Heterogeneity: I^2 = 0%, τ^2 = 0, p = 0.50							
**Platinum dose intensity ≥ 105 mg/m2/3-week = Yes**							
Chen H et al. 2008	20	70	31	70	0.65	[0.41; 1.02]	4.0%
Eddy GL et al. 2007	53	143	51	143	1.04	[0.76; 1.41]	6.5%
Sardi JE et al. 1998	30	74	33	74	0.91	[0.62; 1.32]	5.2%
Sardi JE et al. 1997	19	103	32	103	0.59	[0.36; 0.98]	3.5%
Sardi JE et al. 1996	25	54	41	54	0.61	[0.44; 0.84]	6.2%
**Random effects model**		**444**		**444**	**0.76**	**[0.60; 0.97]**	**25.5%**
Heterogeneity: I^2 = 50%, τ^2 = 0.0376, p = 0.09							
**Random effects model**		**1528**		**1528**	**0.99**	**[0.89; 1.10]**	**100.0%**
Heterogeneity: I^2 = 42%, τ^2 = 0.0230, p = 0.03							
Residual heterogeneity: I^2 = 16%, p = 0.26							
