# Supplementary material for: Dose-Intense Cisplatin-Based Neoadjuvant Chemotherapy Increases Survival in Advanced Cervical Cancer: An Up-to-Date Meta-Analysis
Source: Cancers (Basel). 2022 Feb 8;14(3):842. doi: 10.3390/cancers14030842 (PMC8834199; doi:10.3390/cancers14030842)
Supplement: Supplementary file 1 [file cancers-14-00842-s001.zip › cancers-1512614-Supplementary Table.pdf]

Supplementary Table

Supplementary Table S1: Eligibility using the PICOS criteria

Criteria	Inclusion	Exclusion
Population	Women aged ≥ 18 years with cervical cancer<br>FIGO stages IB2-IVA	FIGO stages IA to IB1<br>FIGO IVB
Intervention	Cisplatin-based neoadjuvant chemotherapy<br>Local treatment using chemoradiation therapy, or radiotherapy, or surgery	Absence of experimental arm using neoadjuvant chemotherapy
Outcomes	Overall survival<br>Progression-free survival	Other outcomes
Study design	Randomized clinical trials with full-texts published results of cisplatin-based neoadjuvant chemotherapy	Retrospective studies<br>Non-randomized trials<br>Meta-analysis<br>Case reports<br>Reviews<br>Conference reports

FIGO, International Federation of Gynecology and Obstetrics

Supplementary table S2: Articles excluded at full-text screening stage

Articles exclusion	Exclusion
Gong L, Zhang J-W, Yin R-T, et al: Safety and Efficacy of Neoadjuvant Chemotherapy Followed by Radical Surgery Versus Radical Surgery Alone in Locally Advanced Cervical Cancer Patients. Int J Gynecol Cancer Off J Int Gynecol Cancer Soc 26:722–728, 2016	Retrospective study
Gupta S, Maheshwari A, Parab P, et al: Neoadjuvant Chemotherapy Followed by Radical Surgery Versus Concomitant Chemotherapy and Radiotherapy in Patients With Stage IB2, IIA, or IIB Squamous Cervical Cancer: A Randomized Controlled Trial. J Clin Oncol Off J Am Soc Clin Oncol 36:1548–1555, 2018	Not cisplatin-based regimen
Marana HRC, de Andrade JM, Dos Reis FJC, et al: Impact of surgical staging in locally advanced cervical cancer and subsequent chemotherapy. J Surg Oncol 100:505–510, 2009	Not compared NACT vs local treatment
Ohara K, Tsunoda H, Tanaka YO, et al: Explanation for the failure of neoadjuvant chemotherapy to improve outcomes after radiotherapy for locally advanced uterine cervical cancer from the standpoint of the tumor regression rate. Radiat Med 25:53–59, 2007	Not randomized trial
Duenas-Gonzalez A, Lopez-Graniel C, Gonzalez-Enciso A, et al: Concomitant chemoradiation versus neoadjuvant chemotherapy in locally advanced cervical carcinoma: results from two consecutive phase II studies. Ann Oncol Off J Eur Soc Med Oncol 13:1212–1219, 2002	Not randomized trial
Benedetti-Panici P, Greggi S, Scambia G, et al: Long-term survival following neoadjuvant chemotherapy and radical surgery in locally advanced cervical cancer. Eur J Cancer Oxf Engl 1990 34:341–346, 1998	Not randomized trial
Kumar L, Kaushal R, Nandy M, et al: Chemotherapy followed by radiotherapy versus radiotherapy alone in locally advanced cervical cancer: a randomized study. Gynecol Oncol 54:307–315, 1994	Duplicate trial

Yuan M, Wang Y, Kou L, et al: Neoadjuvant Chemotherapy Followed by Radical Surgery Versus Concurrent Chemoradiotherapy in Patients With Stage IIA-IIIA Cervical Carcinoma. Int J Radiat Oncol Biol Phys 108:e465–e466, 2020	Retrospective study
